# Effect of Roxadustat on Cardiometabolism in Healthy Individuals (ROXACardioMeta): Protocol for a Double-Blind, Placebo-Controlled and Randomised Cross-Over Trial

**DOI:** 10.3390/mps9020051

**Published:** 2026-03-23

**Authors:** Emma Klemola, Joona Tapio, Rasmus I. P. Valtonen, Mikko P. Tulppo, Janne Hukkanen, Peppi Koivunen

**Affiliations:** 1 Research Unit of Extracellular Matrix and Hypoxia, Faculty of Medical Biochemistry and Molecular Biology, University of Oulu, FI-90014 Oulu, Finland; emma.klemola@oulu.fi (E.K.); joona.tapio@oulu.fi (J.T.); 2 Biocenter Oulu, University of Oulu, FI-90014 Oulu, Finland; 3 Research Unit of Biomedicine and Internal Medicine, Faculty of Medicine, University of Oulu, FI-90220 Oulu, Finland; rasmus.valtonen@oulu.fi (R.I.P.V.); mikko.tulppo@oulu.fi (M.P.T.); janne.hukkanen@oulu.fi (J.H.); 4 Medical Research Center Oulu, University of Oulu, Oulu University Hospital, FI-90014 Oulu, Finland

**Keywords:** cholesterol, HIF, HIF-P4H inhibitors, hypoxia, metabolic syndrome, roxadustat

## Abstract

Hypoxia activates hypoxia-inducible factors (HIFs), which regulate genes involved in erythropoiesis, angiogenesis, and metabolism. HIF stability is controlled by oxygen-dependent HIF prolyl 4-hydroxylases (HIF-P4Hs). Pharmacological HIF-P4H inhibitors are approved for the treatment of anaemia in chronic kidney disease (CKD). Beyond erythropoiesis, these drugs have been linked to improved lipid profiles in CKD, and preclinical studies suggest benefits for glucose tolerance and cardiovascular protection. However, cardiometabolic effects of HIF-P4H inhibitors have not been systematically examined in healthy or non-anaemic individuals. This investigator-initiated, double-blind, placebo-controlled, randomised crossover trial evaluates the systemic effects of roxadustat, an orally administered pan-HIF-P4H inhibitor. The study consists of two 10-day study arms separated by a minimum 4-week washout. Participants receive 70 mg of roxadustat or a placebo thrice a week. The primary hypothesis is that roxadustat lowers plasma total cholesterol. Secondary outcomes include changes in LDL cholesterol, triglycerides, insulin sensitivity, glucose tolerance, body composition, 24 h blood pressure, exercise capacity, autonomic cardiovascular regulation, and skeletal muscle microcirculation. Healthy volunteers (*n* = 24) aged 18–40 years will be enrolled. This study will provide insights into the potential of HIF-P4H inhibitors for obesity, dyslipidaemia, insulin resistance, and hypertension, and may inform future therapeutic strategies for metabolic syndrome, type 2 diabetes, and cardiovascular disease.

## 1. Introduction

A decrease in oxygen concentration leads to hypoxia, which activates a physiological response mechanism. Hypoxia-inducible factor (HIF) is the central transcription factor in this response [1]. Through its target genes, HIF regulates numerous biological pathways, including erythropoiesis and iron metabolism, angiogenesis, energy metabolism, and inflammatory responses [1]. HIF is regulated by three oxygen-dependent HIF prolyl 4-hydroxylase enzymes (HIF-P4Hs 1-3) [1]. The HIF response can be pharmacologically activated by small-molecule HIF-P4H inhibitors [1,2]. The first clinical HIF-P4H inhibitor, roxadustat (Evrenzo), was approved by the European Medicines Agency in August 2021 for the treatment of severe anaemia caused by chronic kidney disease (CKD). To date, clinical trials investigating roxadustat have primarily involved anaemic CKD patients, focusing on its erythropoietic effects and safety [3,4,5,6,7,8,9,10]. Roxadustat is used in both dialysis-dependent (CKD-D) and non-dialysis-dependent (CKD-ND) patients. In addition to its erythropoietic properties, HIF-P4H inhibitors have shown favourable metabolic effects on blood lipid profiles in CKD and peritoneal dialysis patients, lowering total cholesterol, low-density lipoprotein (LDL) cholesterol and triglyceride levels; an effect differentiating them from classical erythropoiesis-stimulating agents, like erythropoietin (EPO) [3,4,10]. Roxadustat and other HIF stabilisers are classified as prohibited substances by the World Anti-Doping Agency (WADA) under category S2: peptide hormones, growth factors, related substances, and mimetics [11].

Beyond CKD patients, HIF-P4H inhibitors have improved blood lipid profiles in animal models. They also reduce adipose tissue mass and inflammation, improve glucose tolerance, and decrease insulin resistance and liver fat accumulation [12,13]. In preclinical studies, pharmacological HIF-P4H inhibition and genetic silencing of *HIF-P4H-2*, the most abundant isoenzyme, have shown benefits in myocardial ischemia and atherosclerosis [14,15,16,17,18]. Roxadustat has also been reported to lower systolic blood pressure in preclinical models [18,19]. HIF target genes such as *NOS3*, *TIE2*, and *APJ* regulate small vessel calibre, and silencing of *HIF-P4H-2* has been shown to enlarge cardiac and skeletal muscle capillaries [15,20]. *MCT4*, which regulates lactate export, is also a HIF target gene, and its upregulation via *HIF-P4H-2* silencing in mice enhances lactate clearance and reuse, protecting against lactic acidosis [20,21].

Despite active research in cell and animal models, the effects of HIF-P4H inhibitors outside their primary erythropoietic indication, particularly in cardiometabolic diseases or among healthy study populations, have not been investigated in clinical trials. Therefore, this study will generate new insights into the potential of HIF-P4H inhibition to influence key metabolic and cardiovascular parameters. These findings may be relevant to the development, prevention, and treatment of obesity, dyslipidaemia, insulin resistance, atherosclerosis, and hypertension. In the future, HIF-P4H inhibitors may potentially become part of the pharmacological treatment for metabolic syndrome, type 2 diabetes, and cardiovascular diseases.

The aim of this study is to investigate the effects of the HIF-P4H inhibitor roxadustat on metabolism and cardiovascular function in healthy volunteers. The primary hypothesis of the study is that roxadustat lowers plasma total cholesterol levels. The secondary hypotheses are that roxadustat lowers plasma LDL cholesterol and triglyceride levels, reduces insulin resistance and improves glucose tolerance in a 2 h oral glucose tolerance test (OGTT), lowers 24 h blood pressure, improves exercise tolerance and autonomic regulation of cardiovascular function, enhances skeletal muscle microcirculation and has a favourable effect on body composition.

## 2. Experimental Design

### 2.1. Trial Design

The study is double-blind, placebo-controlled, and crossover in design. It consists of two 10-day study arms separated by a washout period of at least 4 weeks. The study plan involves recruiting 24 healthy volunteers and administering 70 mg of roxadustat orally (Evrenzo 70 mg, Astellas Pharma (Tokio, Japan), encapsulated in a white capsule) thrice a week over a 10-day period (a total of 4 doses). The effects will be compared to a matched placebo arm, during which participants will receive white placebo capsules thrice a week. Participants are randomised to a treatment sequence. Half of the participants will be randomised to receive roxadustat during study arm 1 and placebo during arm 2, while the other half will receive placebo in arm 1 and roxadustat in arm 2. The randomization will be conducted by the hospital pharmacy of Oulu University Hospital, which holds the randomization code. The code will be unblinded at the end of the study, at which point the pharmacy will provide the allocation information to the principal investigator. At the end of each study arm, participants will undergo blood sampling, a 2 h OGTT, 24 h ambulatory blood pressure monitoring, anthropometric and body composition measurements, heart rate variability assessment, skeletal muscle microcirculation measurement, and a maximal exercise test.

The study will be conducted at the Research Unit of Internal Medicine in the Oulu University Hospital, Oulu, Finland, the Physiology Research Laboratory in the Research Unit of Biomedicine and Internal Medicine, Faculty of Medicine, University of Oulu, and the Faculty of Biochemistry and Molecular Medicine, University of Oulu.

### 2.2. Participants

#### 2.2.1. Inclusion and Exclusion Criteria

Inclusion criteria are healthy volunteers (both women and men, no gender ratio has been stipulated) aged 18–40 years confirmed by medical history, a clinical examination conducted by a physician, and safety laboratory tests (Complete blood count (CBC), plasma potassium, sodium, creatinine, ALT, AFOS, bilirubin, TSH, glucose, hCG (women) from a fasted sample, and urine chemical screening) including an electrocardiogram (ECG)). Eligible participants must have a body mass index (BMI) over 20 kg/m^2^ and weigh less than 100 kg, which is the upper limit defined by the pharmaceutical company (Astellas Pharma) for administering 70 mg of Evrenzo (roxadustat) three times per week.

Exclusion criteria include regular medication use (including continuous allergy medication, thyroxine, and asthma medication, but excluding hormonal intrauterine devices), significant illnesses as assessed by the study physician, systolic blood pressure over 150 mmHg, haemoglobin (Hb) levels outside the Finnish reference ranges (134–167 g/L for men and 117–155 g/L for women), pregnancy and breastfeeding, needle phobia or history of difficult blood sampling, lack of Finnish language proficiency, substance or alcohol abuse, drug use, participation in another clinical trial within one month prior to study start, major surgery within six months prior to study start, allergy to peanuts or soy (the drug coating contains soy), history of venous thrombosis or seizures, significant familial predisposition to thrombosis, hereditary galactose intolerance, complete lactase deficiency, or glucose-galactose malabsorption. Women of childbearing age who are not sterilised or do not use an intrauterine device (IUD) or hormonal IUD during and for one week after the study periods are also excluded. The study is conducted by research nurses and physicians experienced in performing clinical trials and clinical drug studies, and are well-versed in the principles and practices of Good Clinical Practice (GCP). The study is monitored by a clinical trial monitor external to the research groups involved.

#### 2.2.2. Criteria for Discounting Interventions

Criteria for discontinuing an individual participant from the study include withdrawal of informed consent by the participant, any medical condition that, in the opinion of the principal investigator, may compromise the participant’s safety if study medication is continued, pregnancy, ineligibility for the study (either arising during the study or retrospectively identified and missed during screening), an adverse event requiring discontinuation of the study medication, significant non-compliance with the study or study procedures (e.g., dosing, study visits), loss to follow-up despite at least three documented attempts to contact the participant.

Study physicians, researchers and study nurses involved in the study inform the principal investigator of any interim findings that may warrant termination of the trial. The principal investigator makes the final decision regarding discontinuation of the study.

#### 2.2.3. Relevant Concomitant Care Permitted or Prohibited During the Trial

During the study arms and for one week following their completion, women of childbearing potential who are not sterilised must use an effective method of contraception (IUD or hormonal IUD). The use of herbal supplements, trace elements, vitamins, licoricey and salty licoricey (salmiakki) products, and energy drinks is prohibited during the study arms. Consumption of coffee and nicotine products/tobacco should be kept as consistent as possible across both study arms. Participants are asked to avoid significant deviations in fat quantity or quality from their usual diet, as well as highly salty foods; otherwise, a normal diet is recommended. Alcohol consumption is prohibited during the study arms. Participants are also asked to refrain from unusually strenuous physical activity during the study arms. Use of anti-inflammatory pain medications is discouraged during the study arms. For occasional pain, paracetamol is recommended. If the need for medication arises during the study, participants are asked to inform the study staff so that the safety of continued participation can be assessed. During the washout period, participants may follow their normal lifestyle.

#### 2.2.4. Sample Size

A total of 24 healthy volunteers will be recruited for the study. If any participants discontinue, new individuals will be recruited to ensure the final sample size remains at 24. The hospital pharmacy will assign the same treatment sequence to the replacement participant as was assigned to the one who withdrew. Data collected from participants who discontinue will not be used. With this sample size, the study has a 95% probability (power) of detecting a 0.3-unit (mmol/L) change in plasma total cholesterol levels at a two-sided significance level of 0.05, assuming a standard deviation (SD) of 0.38 mmol/L for the change in the primary outcome. The assumptions for this power calculation are based on a previous clinical trial evaluating the efficacy of roxadustat, in which a 3-wk treatment reduced plasma total cholesterol by 0.9 mmol/L more in CKD-ND patients (*n* = 82–84) compared to those treated with placebo (*n* = 43), with the between-group difference remaining consistent for up to nine weeks after treatment initiation [3].

#### 2.2.5. Compensations

Each participant will receive a one-time compensation of 120 €, in accordance with Regulation 82/2011, as reimbursement for participating in a medical trial that does not provide direct health benefits to the individual. Participants who complete only the first study arm will receive half of the total compensation. In addition, participants will gain valuable information about their blood values, body composition, blood pressure, circulation, autonomic nervous system function, and aerobic fitness.

If a personal injury occurs due to the investigational drug or procedure performed as part of the study, compensation may be sought. Compensation for harm caused by the investigational drug is applied for through the Pharmaceutical Injury Insurance. This insurance covers unexpected adverse effects caused by the investigational drug under conditions specified in the insurance terms. For personal injuries not caused by the investigational drug, compensation is sought through the patient’s insurance or the subject insurance of Oulu University Hospital or the University of Oulu.

#### 2.2.6. Plans to Ensure Complete Follow-Up

All participants will gain valuable information about their blood values, body composition, blood pressure, circulation, autonomic nervous system function, and aerobic fitness. Sharing these data with the participants is utilised to promote retention and ensure completion of the two study arms. Data collected from participants who discontinue will not be used in analyses.

### 2.3. Blinding and Randomization

The study is double-blind. Trial participants, care providers and outcome assessors will be blinded during the data collection. Randomization will be performed for the treatment sequence, and it is managed by the hospital pharmacy at Oulu University Hospital, which holds the randomization code. Half of the participants will be randomised to receive roxadustat during study arm 1 and placebo during arm 2, while the other half will receive placebo during arm 1 and roxadustat during arm 2. The pharmacy delivers the drugs in envelopes having the participant number (1–24) and study arm (1 or 2) to the research centre, where they are kept at room temperature in a locked cabinet. Any unused drugs are returned to the pharmacy, which is responsible for disposal. Both the study medicine (Roxadustat, Evrenzo 70 mg) and the placebo are packed in similar, white-coloured capsules. The code will be blinded until the end of the study (i.e., until 24 participants have completed two study arms), at which point the pharmacy will provide the allocation information to the principal investigator. In case of serious adverse events or pregnancy, the randomization code will be unblinded immediately.

### 2.4. Data Monitoring

Due to the nature of the trial (small number of healthy volunteers and short study duration), there is no data monitoring committee. Monitoring of the study is conducted by a risk-based approach. The principal investigator has assessed the level of monitoring using a three-tier scale, placing the study in the medium-risk category based on risk-based evaluation. This assessment considers the investigational product/placebo, the duration and nature of the intervention, as well as the number and characteristics of the study subjects. Level 2 monitoring includes the informed consent process, the investigator’s study file, serious adverse events, subject discontinuations and withdrawals, inclusion and exclusion criteria, and evaluation and verification of the primary endpoints on a per-subject basis. The proper management and handling of the investigational product/placebo are also verified. In addition, source data verification (SDV) is performed 100% for the first two subjects and thereafter for every third subject. The monitoring also includes a site initiation visit before the start of the study and a close-out visit after the final study visit. Other monitoring visits are conducted at least every four months. The monitor ensures that the rights and well-being of the subjects are protected, that written informed consent has been obtained from all subjects prior to participation, that the study is conducted in accordance with the approved protocol and applicable regulations, and that the collected study data are accurate and complete. If the monitor identifies any deviations from the study protocol, procedures, GCP, or applicable regulations, they will promptly report these to the study sponsor and the principal investigator and aim to prevent recurrence of the deviations. The study monitoring is conducted by a trained monitor external to the research groups involved in the trial. Monitoring visits are recorded in the monitoring log form, and reports of the visits are submitted to the principal investigator. Subjects are provided with sufficient information about the monitoring, both in the subject information sheet and verbally. The subject’s personal data protection may be compromised, as the regulatory authority (the Finnish medicines agency Fimea) has the right to access patient records during inspections.

### 2.5. Adverse Event Reporting and Harms

Adverse events related to the study are monitored by actively inquiring about them during study visits, as well as by reviewing laboratory test results and physiological measurements. Study subjects have the opportunity to report adverse events to the principal investigator around the clock via phone and email. Adverse events and side effects will be recorded, and all adverse reactions will be reported annually to Fimea via secure email. At the same time, a summary of the overall safety of individuals participating in the clinical trial will be submitted. All serious adverse events, except for pulmonary embolism, are considered Suspected Unexpected Serious Adverse Reactions (SUSARs) and must be reported to Fimea as soon as possible, but no later than within 7 days. Any updates to the initial report must be submitted within 8 days of the first notification. These reports will be sent via secure email to Fimea. Fimea will forward the information to EudraVigilance on behalf of the principal investigator. SUSARs that are not life-threatening or fatal must be reported within 15 days. Additionally, a summary of the study results must be submitted to Fimea no later than one year after the study has concluded.

### 2.6. Confidentiality

The research nurses and study physicians participating in the study are well-versed in the principles and practices of GCP. All collected data and samples will be processed using pseudonymized coding; individual data cannot be identified from any publications related to the study. The research data will be handled in accordance with the data protection policies of Oulu University Hospital and the University of Oulu, in compliance with European GDPR legislation. Only researchers involved in the study will have access to the research data, and they are bound by confidentiality.

## 3. Procedure

### 3.1. Recruitment

Recruitment will primarily be based on an announcement distributed via email lists of the Oulu Medical Guild and the Oulu Dental Guild at the Faculty of Medicine, University of Oulu. If necessary, individuals other than medical and dental students may also be recruited; newspaper advertisements will not be used.

### 3.2. Implementation

Individuals who respond to the recruitment announcement will be interviewed by phone using a structured interview form by an investigator. If no concerns arise during the interview regarding eligibility for the study, an appointment will be scheduled for the screening visit. Based on these measurements and assessments, the study physician makes the final decision regarding the individual’s eligibility for participation. The study schedule is reviewed with the individual, and an appointment for the first study visit is booked.

### 3.3. Intervention Description

Study Day 1:

- Female participants undergo a urine pregnancy test (Apteekkarin pregnancy test, CE-marked). In case the result is positive, participation in the study is discontinued.
- Weight, height and waist and hip circumference are measured using a scale (Seca Model 861, Seca GmbH, Hamburg, Germany), stadiometer (Seca), and measuring tape, respectively.
- Blood pressure and pulse are measured using an arm cuff monitor (GE CARESCAPE Dinamap V100, GE Healthcare, Chicago, IL, USA).
- Study medication (either placebo or roxadustat capsules, blinded and provided by the hospital pharmacy) is dispensed. The first dose is taken under supervision during the visit; the subsequent doses are taken independently at home on study days 3, 5, and 8.
  ○ Participants record each dose of medication taken in a diary provided during the Day 1 visit and return the empty medication packages at the final visit of the study arm.

Study Day 9 (Morning, participant fasted for 10–12 h):

- Weight, waist and hip circumference are measured on Study Day 1.
- Blood pressure and pulse are measured on Study Day 1.
- Fasting blood samples are collected for the following analyses in the clinical laboratory (NordLab) of the Oulu University Hospital, and results are shared with participants: CBC with Differential, E-Reticulocytes, plasma Potassium, Sodium, Creatinine, ALT, AST, Bilirubin, ALP, gamma-GT, Albumin, TSH, Total Cholesterol, LDL, HDL, Triglycerides, hsCRP, HbA1c, lp(a). Additional analyses may be performed in academic or commercial labs.
- Additional blood samples (2 × 8 mL) are collected in CPT tubes (BD Biosciences, San Jose, CA, USA) for isolation of mononuclear cells and RNA extraction (RNeasy Mini Kit, Qiagen, Hilden, Germany) to study gene expression related to HIF response and drug metabolism. DNA is also extracted from the blood sample, enabling the potential to investigate genes that could be involved in glucose, lipid, hormone, bone, and drug metabolism, or blood pressure regulation.
- Serum and plasma samples (10 mL each; plasma with EDTA) are stored for potential additional analyses related to metabolism, including serum metabolomics and plasma 4β-hydroxycholesterol levels. If the participant withdraws, these samples will be destroyed.
- After blood sampling, a 2 h OGTT is performed. The participant drinks a solution containing 75 g of glucose. Venous blood samples are taken at 0, 30, 60, and 120 min to measure glucose, insulin, and C-peptide in Nordlab. These results are shared with the participants. At each time point, 2 × 5 mL blood samples are collected, immediately treated with a dipeptidyl peptidase IV (DPP) inhibitor, and used for glucagon, GLP-1 and GIP assays by ELISA at the Faculty of Biochemistry and Molecular Medicine, University of Oulu. One sample is stored for potential further analyses.
- Participants are instructed in the use of an ambulatory blood pressure monitor (Mobil-O-Graph, IEM GmbH, Aachen, Germany), which is worn for 24 h at home. Data is analysed using HMS-CS Hypertension Management Software, version 6.3 (IEM) [22].

Study Day 10 (Afternoon):

- Participant returns the ambulatory blood pressure monitor.
- Body composition measurements:
  ○ Bioimpedance technique is used to measure lean mass, soft tissue mass, fat mass, total body water, and visceral fat by the InBody 720 device (InBody Co., Ltd., Seoul, Republic of Korea).
  ○ Skinfold thickness is measured from the thigh and arm (near-infrared spectroscopy [NIRS] sensor area) using a Harpenden calliper (Baty International, UK).
- Autonomic nervous system regulation assessment:
  ○ Heart rate variability, blood pressure variability, and respiratory rate are measured at rest in a seated position for 5 min:
    ▪ ECG: 3-channel ECG (MAC 5500 HD, GE Healthcare, Chicago, IL, USA)
    ▪ Blood pressure: Non-invasive finger cuff (Nexfin, BMEYE Medical Systems, Amsterdam, The Netherlands)
    ▪ Respiratory rate: PneumoTrace (ADInstruments, Sydney, Australia) [23]
- Assessment of microcirculation:
  ○ A NIRS device (Oxymon MK III, Artinis Medical Systems, Elst, The Netherlands), assessing changes in oxyhaemoglobin, deoxyhaemoglobin, total haemoglobin, and tissue saturation index (TSI), is used to measure skeletal muscle microcirculation non-invasively at rest and during occlusion [24,25].
  ○ Measurements are taken at rest (1 min), during occlusion (5 min), and recovery (3 min) from the forearm (*M. flexor digitorum superficialis*).
  ○ Occlusion is performed using a rapid-inflation cuff (Hokanson model E20, Hokanson, Inc., Bellevue, WA, USA) on the left arm.
  ○ A skin map is drawn based on skin markings to ensure consistent measurement location in the second study arm.
- Maximal exercise test (VO_2_max):
  ○ Conducted using a cycle ergometer (Monark Ergomedic 839E, Vansbro, Sweden) starting at 40 W with ramp protocol (men: +20 W/min, women: +15 W/min), and gas exchange was monitored (Vyntus™ CPX, Vyaire Medical, Chicago, IL, USA)
  ○ Measurements during rest, exercise, and recovery include blood pressure, 15-lead ECG, heart rate, blood lactate, and muscle microcirculation:
    ▪ Blood pressure: Measured at rest, during each load stage, and post-exercise (2, 5 min and 10 min) using an arm cuff (Schiller BP-200-plus, Schiller, Doral, FL, USA; Korotkoff sound analysis with QRS trigger).
    ▪ ECG: Continuous 15-lead ECG (CAM-14; GE Healthcare, Dusseldorf, Germany).
    ▪ Heart rate: Monitored continuously using Polar Verity Sense and Polar H10 chest strap (Polar Electro, Kempele, Finland).
    ▪ Blood lactate: Measured from fingertip using Lactate Scout+ metre (SensLab/EKF Diagnostics, Leipzig, Germany) at rest, every 2 min during exercise, and post-exercise (5 min and 10 min).
    ▪ Microcirculation: Continuously measured using NIRS (Artinis Medical Systems, The Netherlands) from the outer thigh muscle (*M. vastus lateralis*).

**Figure 1:**
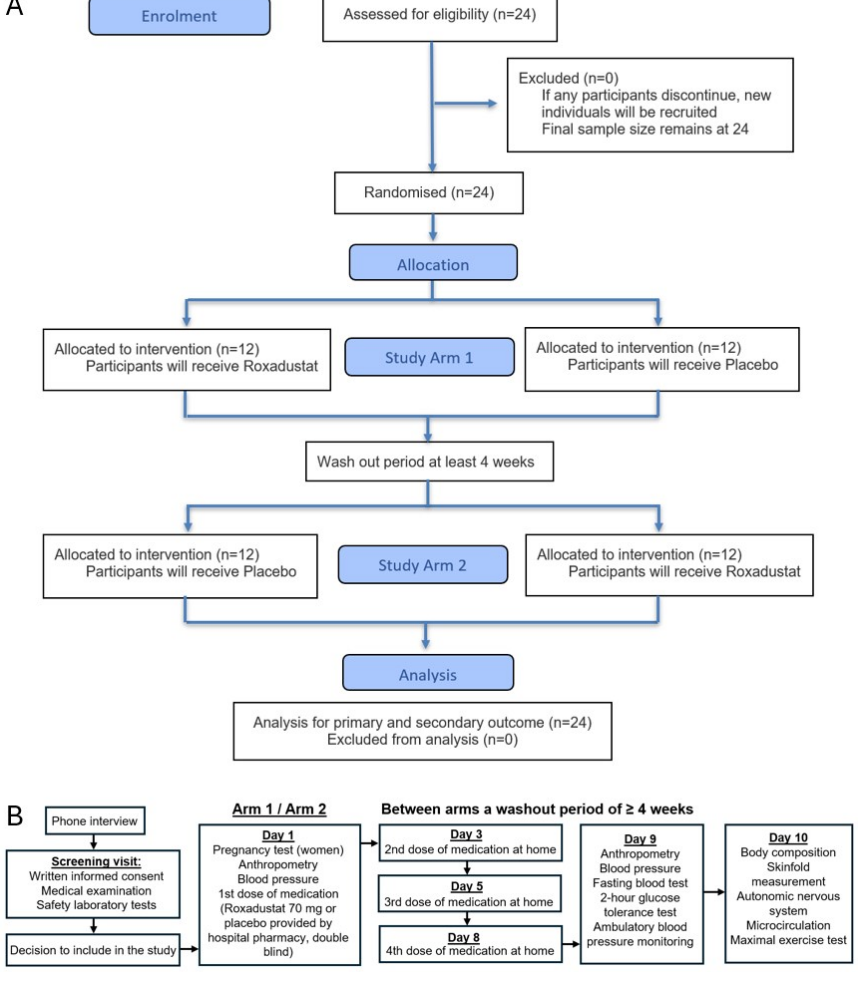
Flow diagram (**A**) and participant flow chart (**B**).

**Figure 2:**
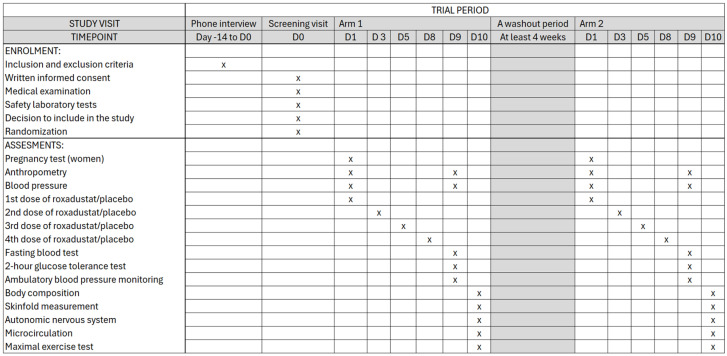
Schedule of enrolment, interventions and assessment.

The flow diagram and participant flow chart are described in Figure 1, and the schedule of enrolment, interventions and assessments in Figure 2.

## 4. Expected Results

### 4.1. Primary and Secondary Outcomes

The primary outcome is the change in plasma total cholesterol levels. The secondary outcomes are changes in plasma LDL cholesterol and triglycerides levels, insulin resistance and glucose tolerance in a 2 h OGTT, 24 h blood pressure, exercise tolerance and autonomic regulation of cardiovascular function, skeletal muscle microcirculation and body composition. All measurements will be conducted during both study arms. Results obtained during the study arm in which participants receive roxadustat will be compared to the results from the placebo arm. Participants will serve as their own controls.

### 4.2. Statistical Analyses

Statistical analyses will be mostly conducted using Student’s paired (dependent samples) *t*-test, as each participant serves as their own control in a crossover study design, and the long interval between study arms is assumed to eliminate any sequence effect. The normality of the measured variables will be assessed. If a variable is not normally distributed, a logarithmic transformation will be applied prior to statistical analysis, or non-parametric tests will be used. For multivariable analyses, regression models will be employed as the statistical method. Principal component analysis will be used to account for multiple testing when necessary. Sensitivity analyses on potential period or carry-over effects will be conducted.

### 4.3. Data Management

The research results will be archived and stored at the Oulu University Hospital and the University of Oulu after the study has ended. The retention period for study documents is 25 yrs after the end of the trial.

## 5. Discussion

This double-blind, placebo-controlled and randomised trial provides a unique opportunity to explore the systemic effects of the HIF-P4H inhibitor roxadustat in healthy individuals. While roxadustat is currently approved for the treatment of anaemia associated with CKD, its broader metabolic and cardiovascular effects remain largely uncharacterised in clinical settings. To date, clinical trials have primarily focused on anaemic CKD patients, both CKD-D and CKD-ND, with the main emphasis on erythropoietic efficacy and safety [3,4,5,6,7,8]. Lately, erythropoietic effects have also been shown in other patient groups, including diabetic ND-CKD patients [26], aplastic anaemia [27] and patients receiving chemotherapy for non-myeloid malignancies [28]. A recent study on end-stage kidney disease patients reported a 6-month roxadustat treatment to increase markers of calcification [29]. This, and other potential risks associated with the treatment of patients with roxadustat and other drugs of the same class, should be taken into account [30].

Emerging evidence from these studies suggests that HIF-P4H inhibitors may exert favourable metabolic effects beyond erythropoiesis. In CKD and peritoneal dialysis patients, roxadustat has been associated with reductions in total cholesterol, LDL cholesterol, and triglyceride levels [3,9,10]. These findings, although promising, have largely been observed as secondary outcomes and have not yet been systematically evaluated in non-anaemic or healthy individuals.

Beyond CKD patients, HIF-P4H inhibitors have demonstrated metabolic benefits in animal models, including improved blood lipid profiles, reduced adipose tissue mass and inflammation, enhanced glucose tolerance, and decreased insulin resistance and hepatic fat accumulation [11,12]. Preclinical studies have also shown that both pharmacological inhibition and genetic silencing of HIF-P4H-2, the most abundant isoenzyme, confer protection in models of myocardial ischemia and atherosclerosis [13,14,15,16,17], further supporting its potential cardiovascular benefits. Interestingly, a recent study on haemodialysis patients found roxadustat more helpful in regression of left ventricle hypertrophy compared to EPO [31].

The aim of this study is to investigate the effects of roxadustat on metabolism and cardiovascular function in healthy individuals. By evaluating diverse endpoints, the study seeks to provide a comprehensive understanding of the physiological impact of HIF-P4H inhibition beyond its erythropoietic role. If roxadustat demonstrates beneficial effects without significant adverse outcomes, it could pave the way for new therapeutic applications (drug repurposing) in the prevention and treatment of metabolic syndrome, type 2 diabetes, and cardiovascular diseases. Furthermore, the findings may offer novel insights into the role of hypoxia-inducible factor signalling in regulating metabolic and cardiovascular homeostasis in humans.

## Data Availability

The authors of this manuscript have access to the final data set. No plans for granting public access to the full protocol, participant-level dataset or statistical code. The Finnish law stipulates that no health-related individual data can be shared with non-study persons. Clustered data (with a minimum of five volunteers clustered) can be shared on a reasonable request.

## Supplementary Materials

The following supporting information can be downloaded at: https://www.mdpi.com/article/10.3390/mps9020051/s1, Documents: Approval of the trial by the Finnish Medicines Agency (Fimea) and National Committee on Medical Research Ethics (Tukija); decision date 12 April 2024 (Reference number FIMEA/2024/002165), and English translation of the consent to participate.

## Abbreviations

The following abbreviations are used in this manuscript:

ALP	Alkaline Phosphatase
ALT	Alanine Aminotransferase
APJ	Apelin Receptor
AST	Aspartate Aminotransferase
BMI	Body Mass Index
CBC	Complete Blood Count
CKD	Chronic Kidney Disease
CKD-D	Dialysis-Dependent Chronic Kidney Disease
CKD-ND	Non-Dialysis-Dependent Chronic Kidney Disease
CPT	Cell Preparation Tube
DNA	Deoxyribonucleic Acid
DPP	Dipeptidyl Peptidase IV
ECG	Electrocardiogram
EDTA	Ethylenediaminetetraacetic Acid
EPO	Erythropoietin
Fimea	Finnish Medicines Agency
GCP	Good Clinical Practice
GDPR	General Data Protection Regulation
GIP	Gastric Inhibitory Polypeptide
GLP-1	Glucagon-Like Peptide-1
HbA1c	Haemoglobin A1c
HDL	High Density Lipoprotein
HIF	Hypoxia-Inducible Factor
HIF-P4H	HIF Prolyl 4-Hydroxylases
hsCRP	High-Sensitivity C-Reactive Protein
i.e.,	Id Est
IUD	Intrauterine Device
K	Kalium
LDL	Low Density Lipoprotein
lp(a)	Lipoprotein A
MCT4	Monocarboxylate Transporter 4
Na	Natrium
NIRS	Near-Infrared Spectroscopy
NOS3	Nitric Oxidase Synthetase 3
OGTT	Oral Glucose Tolerance Test
RNA	Ribonucleic Acid
SD	Standard Deviation
SDV	Source Data Verification
SUSARs	Suspected Unexpected Serious Adverse Reactions
TIE2	Tyrosine Kinase With Immunoglobulin-Like And EGF-Like Domains 2
TSH	Thyroid-Stimulating Hormone
TSI	Tissue Saturation Index
WADA	World Anti-Doping Agency
