# Supplementary material for: Effect of Roxadustat on Cardiometabolism in Healthy Individuals (ROXACardioMeta): Protocol for a Double-Blind, Placebo-Controlled and Randomised Cross-Over Trial
Source: Methods Protoc. 2026 Mar 23;9(2):51. doi: 10.3390/mps9020051 (PMC13118563; doi:10.3390/mps9020051)
Supplement: Supplementary file 1 [file mps-09-00051-s001.zip › mps-4123656-supplementary.pdf]

**Supplementary Material**

**Effect of Roxadustat on Cardiometabolism in Healthy Individuals (ROXACardioMeta): Protocol for a double-blind, placebo-controlled and randomised cross-over trial**

Emma Klemola[1,2], Joona Tapio[1,2], Rasmus Valtonen[3,4], Mikko P. Tulppo[3,4], Janne Hukkanen[3,4 *], Peppi Koivunen[1,2*]

[1]Research Unit of ECM & Hypoxia, Faculty of Medical Biochemistry and Molecular Biology, University of Oulu, [2]Biocenter Oulu, University of Oulu, Finland, [3]Research Unit of Biomedicine and Internal Medicine, Faculty of Medicine, University of Oulu, [4]Medical Research Center Oulu, University of Oulu and Oulu University Hospital.

Content:

Original and English translation of Ethics approval by The Finnish Medicines Agency (Fimea) and National Committee on Medical Research Ethics (Tukija) (Reference number FIMEA/2024/002165).

English translation of consent to participate.

**Päätös**

12.4.2024

Dnro FIMEA/2024/002165
CTIS 2023-508183-29-00

Pohjois-Pohjanmaan hyvinvointialue
Janne Hukkanen
Kajaanintie 50
90220 Oulu

## LUPA KLIINISEN LÄÄKETUTKIMUKSEN SUORITTAMISELLE TIETYIN EDELLYTYKSIN

### Hakemus

Pohjois-Pohjanmaan hyvinvointialue (Janne Hukkanen) on hakenut lupaa seuraavalle kliiniselle lääketutkimukselle:

*Effect of Roxadustat on Cardiometabolism*

### Ratkaisu

Lääkealan turvallisuus- ja kehittämiskeskus Fimea on päättänyt myöntää luvan hakemuksessa tarkoitetulle kliiniselle lääketutkimukselle seuraavin edellytyksin:

Hakemuksen osa II:

Edellytys 1

Regarding the RFI-CT-2023-508183-29-00-IN-003-03 and RFI-CT-2023-508183-29-00-IN-003-06, the changes made are insufficient. The information sheet and informed consent form need further revision. This condition is to be fulfilled via Non-Substantial Modification (NSM).
More specifically, in the section "Pyyntö osallistua tutkimukseen", instead of the term 'lääke', 'tutkimuslääke' should be used (…-lääkkeen vaikutuksia…--> …-tutkimuslääkkeen vaikutuksia…). On page 3, the phrase "nautittava lääke" should be changed to, for example, following: "otettava tutkimuslääke". The phrase "seerumi- ja plasmafraktioista" (p. 4/9) should be explained briefly or alternative, easier-to-understand phrase should be employed. The same applies to the consent form phrases "seerumin metabolomiikka" and "plasman 4beta-hydroksikolesteroli". Required methods of contraception for men needs to be specified in the information sheet. The section "Tutkimuksen mahdolliset hyödyt" should begin with the following or a corresponding phrase: "On mahdollista, ettei tähän tutkimukseen osallistumisesta ole hyötyä tutkittaville". In the section "Tutkimuksesta mahdollisesti aiheutuvat haitat ja epämukavuudet", the following text "Tässä tutkimuksessa lääkkeen käyttö on lyhytaikainen (10 päivää, 4 annosta) ja seuranta tiivistä ja tutkittavat ovat terveitä vapaaehtoisia, joten merkittävien haittavaikutusten riski on vähäinen. Odotettavat terveysvaikutukset ovat lyhyen hoitojakson vuoksi pieniä" should be moved to the beginning. Hyperkalemia (p. 6) should be explained in brackets or an alternative easier-to-understand term should be used. All imperative formulations should be removed (e.g. "otatte" -> teitä pyydetään ottamaan/teidän tulee ottaa).

Tutkimuksen voi aloittaa, mutta muutoksia vaativia dokumentteja ei voi käyttää tutkimuksessa, ennen kuin muutetut dokumentit on lähetetty CTIS-portaaliin (NSM-muutoksena).

**Perustelut**

Suomi on toiminut hakemuksen arvioinnissa raportoivana jäsenvaltiona. Fimea ja Valtakunnallinen lääketieteellinen tutkimuseettinen toimikunta (Tukija) ovat arvioineet hakemuksen yhteistyössä kliinisestä lääketutkimuksesta annetun lain (983/2021) 7 §:ssä säädetyn menettelyn mukaisesti.

Arviointiraportin I osaan kuuluvat seikat

Fimea ja Tukija ovat arvioineet hakemuksen EU:n lääketutkimusasetuksen (536/2014) 6 artiklassa mainittujen seikkojen osalta ja katsonut hakemuksen olevan hyväksyttävissä mainitussa asetuksessa vahvistettujen vaatimusten perusteella.

Tarkempien hakemuksen I osan hyväksyttävyyttä koskevien perustelujen osalta viitataan arviointiraportin I osaan.

Arviointiraportin II osaan kuuluvat seikat

Tukija on arvioinut hakemuksen EU:n lääketutkimusasetuksen 7 artiklassa mainittujen seikkojen osalta ja katsonut, että hakemus on hyväksyttävissä EU:n lääketutkimusasetuksessa ja sen nojalla annetussa kansallisessa lainsäädännössä vahvistettujen vaatimusten perusteella, mutta siihen sovelletaan erityisehtoja, jotka on lueteltu arviointiraportin II osan päätelmässä.

Tarkempien hakemuksen II osan hyväksyttävyyttä koskevien perustelujen osalta viitataan arviointiraportin II osaan.

**Sovelletut säännökset**

Euroopan parlamentin ja neuvoston asetus (EU) N:o 536/2014 ihmisille tarkoitettujen lääkkeiden kliinisistä lääketutkimuksista ja direktiivin 2001/20/EY kumoamisesta, artiklat 4, 5, 6, 7 ja 8

Laki kliinisestä lääketutkimuksesta (983/2021) 7 §, 11 § ja 12 §

**Allekirjoitus**

Piia Vuorela
johtaja

Elina Rantala
ylilääkäri

*Tämä asiakirja allekirjoitetaan sähköisesti. Allekirjoitus on tarkistettavissa erillisestä allekirjoitussivusta.*

fimea

EU:n lääketutkimusasetuksen 8 artiklan 9 kohdan mukaan, jos tutkimukseen ei ole otettu tutkittavia Suomesta kahden vuoden kuluessa tämän luvan myöntämisestä, lupa raukeaa Suomessa, paitsi jos määräaikaa on toimeksiantajan pyynnöstä pidennetty mainitun asetuksen luvussa III säädettyä menettelyä noudattaen.

**Päätöksen muutoksenhakukelpoisuus**

EU:n lääketutkimusasetuksen 8 artiklan perusteella tähän päätökseen ei ole mahdollista hakea muutosta.

**Käsittelymaksu**

Hakemus on käsitelty maksutta kliinisiä lääketutkimuksia koskevista maksullisista suoritteista annetun sosiaali- ja terveysministeriön asetuksen (103/2022) 3 §:n 1 kohdan nojalla.

**Lisätiedot**

Lisätietoja asiassa antaa tarvittaessa:

Elina Rantala, +358 29522 3383, elina.rantala@fimea.fi

clinicaltrials@fimea.fi

**Asiakirjan sähköinen allekirjoitus**
**Elektronisk underskrift av dokument**
**Electronic signature of a document**

**Asia / Ärende / Case:**
FIMEA/2024/002165
EU CT 2023-508183-29-00

**Asiakirja / Dokument / Document:**
FIMEA/2024/002165-1
EU CT 2023-508183-29-00 FI decision.docx

**Allekirjoitukset / Underskrifter / Signatures:**

Signed By:Piia Vuorela
Signed at:2024-04-12 09:01:00 +03:00
Reason:Witnessing Piia Vuorela

Signed By:Elina Rantala
Signed at:2024-04-12 06:37:13 +03:00
Reason:Witnessing Elina Rantala

fimea

**Decision**

12.4.2024

Ref. no. FIMEA/2024/002165

CTIS 2023-508183-29-00

Northern Ostrobothnia Welfare Region
Janne Hukkanen
Kajaanintie 50
90220

**PERMIT TO CONDUCT CLINICAL MEDICAL RESEARCH UNDER CERTAIN CONDITIONS**

**Applicati on**

The Northern Ostrobothnia Welfare Region (Janne Hukkanen) has applied for a permit for the following clinical drug trial:

*Effect of Roxadustat on Cardiometabolism*

**Decision**

The Finnish Medicines Agency Fimea has decided to grant a permit for the clinical drug trial referred to in the application under the following conditions:

Part II of the application:

Condition 1

Regarding the RFI-CT-2023-508183-29-00-IN-003-03 and RFI-CT-2023-508183-29-00-IN-003-06, the changes made are insufficient. The information sheet and informed consent form need further revision. This condition is to be fulfilled via Non-Substantial Modification (NSM).
More specifically, in the section "Request to participate in the study", instead of the term 'medicine', 'investigational medicine' should be used (…-effects of the medicine…-
-> …-effects of the investigational drug…). On page 3, the phrase "drug taken orally" should be changed to, for example, the following: "investigational drug taken orally" The phrase "serum and plasma fractions" (p. 4/9) should be explained briefly or an alternative, easier-to-understand phrase should be used. The same applies to the consent form phrases "serum metabolomics" and "plasma 4beta-hydroxy cholesterol". Required methods of contraception for men need to be specified in the information sheet. The section "Possible benefits of the study" should begin with the following or a corresponding phrase: "It is possible that participation in this study will not be beneficial to the subjects." In the section "Possible harms and discomforts of the study," the following text "In this study, the use of the drug is short-term (10 days, 4 doses) and monitoring is intensive, and the subjects are healthy volunteers, so the risk of significant side effects is low.
The expected health effects are minor due to the short treatment period" should be moved to the beginning. Hyperkalemia (p. 6) should be explained in brackets or an alternative, easier-to-understand term should be used. All imperative formulations should be removed (e.g., "you will take" -> you are asked to take/you must take).

fimea

The study can be started, but documents requiring changes cannot be used in the study until the amended documents have been sent to the CTIS portal (as an NSM change).

**Justification**

Finland has acted as the reporting Member State in the evaluation of the application. Fimea and the National Committee on Medical Research Ethics (Tukija) have evaluated the application in cooperation in accordance with the procedure laid down in Section 7 of the Act on Clinical Medicinal Research (983/2021).

Part I of the assessment report covers the following points

Fimea and Tukija have assessed the application with regard to the matters referred to in Article 6 of the EU Clinical Trials Regulation (536/2014) and considered the application to be acceptable on the basis of the requirements laid down in that Regulation.

For more detailed reasons regarding the acceptability of Part I of the application, please refer to Part I of the assessment report.

Points covered in Part II of the assessment report

The sponsor has assessed the application in relation to the aspects mentioned in Article 7 of the EU Clinical Trials Regulation and considered that the application is acceptable on the basis of the requirements laid down in the EU Clinical Trials Regulation and in the national legislation adopted pursuant to it, but that it is subject to the specific conditions listed in the conclusion of Part II of the assessment report.

For more detailed reasons regarding the acceptability of Part II of the application, please refer to Part II of the assessment report.

**Applicable provisions**

Regulation (EU) No 536/2014 of the European Parliament and of the Council on clinical trials on medicinal products for human use and repealing Directive 2001/20/EC, Articles 4, 5, 6, 7, and 8

Act on Clinical Trials (983/2021), Sections 7, 11 and 12

**Signature**

Piia Vuorela
Director

Elina Rantala
Chief Physician

*This document is signed electronically. The signature can be verified on a separate signature page.*

Finnish Medicines Agency | Säkerhets- och utvecklingscentret för läkemedelsområdet | Finnish Medicines Agency PL 55, 00034 FIMEA | Tel. 029 522 3341 |kirjaamo@fimea.fi | Business ID: 0921536-6 | fimea.fi

fimea

According to Article 8(9) of the EU Clinical Trials Regulation, if no subjects from Finland have been enrolled in the trial within two years of the authorisation being granted, the authorisation shall lapse in Finland, unless the deadline has been extended at the request of the sponsor in accordance with the procedure laid down in Chapter III of the Regulation.

**Appealability of the decision**

Pursuant to Article 8 of the EU Clinical Trials Regulation, this decision is not subject to appeal.

**Processing fee**

The application has been processed free of charge pursuant to Section 3(1) of the Ministry of Social Affairs and Health Decree on chargeable services related to clinical trials (103/2022).

**Further**

For further information, please contact:

Elina Rantala, +358 29522 3383, elina.rantala@fimea.fi

clinicaltrials@fimea.fi

**Electronic signature of a document**
**Elektronisk underskrift av dokument**

**Case:**
FIMEA/2024/002165
EU CT 2023-508183-29-00

**Document:**
FIMEA/2024/002165-1
EU CT 2023-508183-29-00 EN decision.docx

**Signatures / Underskrifter / Signatures:**

Signed By: Piia Vuorela
Signed at:2024-04-12 09:01:00 +03:00
Reason:Witnessing Piia Vuorela

Signed By: Elina Rantala
Signed at: 2024-04-12 06:37:13 +03:00
Reason: Witnessing Elina Rantala

## CONSENT TO PARTICIPATE IN A CLINICAL DRUG TRIAL

I have been invited to participate in the clinical drug trial titled “The Effect of Roxadustat on Cardiometabolism in Healthy Individuals” involving the drug Evrenzo (roxadustat) (CTIS number: 2023-508183-29-00). The purpose of the study is to investigate the effects of Evrenzo (roxadustat) on metabolism and the function of the cardiovascular system.

I have received, read, and understood the information sheet describing the study. The sheet has provided sufficient details about the study and the collection, processing, and disclosure of data involved. The contents of the information sheet have also been explained to me orally, and I have received satisfactory answers to all my questions regarding the study. The information was provided by ______________________________________, ___ / ___ 20 ___. I have had sufficient time to consider my participation in the study.

I have been informed about the sources from which my personal data will be collected. I give permission for the collection of data necessary for the study to be stored in the research registry of the Department of Internal Medicine. If required by the study, data may be requested from healthcare providers where my patient records are held. For this purpose, the physician may record my personal identification number and use it to obtain the necessary information. A note of my participation in the study will be added to my patient records at Oulu University Hospital.

All data collected about me during the study will be treated confidentially. The data collected will be coded so that my identity cannot be determined without a decoding key. The decoding key will be stored securely in the principal investigator’s archive.

Representatives of the authority responsible for drug regulation and safety (Fimea) have the right to verify the accuracy of the study data and the proper conduct of the study. This will be done by comparing the study data with my original medical records and health information. All aforementioned parties are obligated to maintain confidentiality.

The data collected in this study may be processed outside the premises and equipment of the investigator who collected the data. In such cases, the data will remain in coded form.

I understand that my participation in this study is entirely voluntary. I have the right to withdraw from the study at any time without providing a reason. Refusing to participate or withdrawing from the study will not affect any medical care I may need now or in the future.

I am aware that data collected about me up to the point of withdrawal will be used as part of the study material. By signing this form, I also consent to being contacted in the future if the researchers wish to invite me to participate in follow-up studies.

By signing below, I confirm my participation in this study and voluntarily consent to be a research subject.

____________________________________ _________________________

Signature Date

____________________________________

Printed Name

Consent received

____________________________________ _________________________

Investigator’s Signature Date

____________________________________

Printed Name

Principal Investigator: Professor Janne Hukkanen, Specialist in Internal Medicine Address and phone number: OYS, Department of Internal Medicine, Kajaanintie 50, 90220 Oulu, 08-315 6212

The original signed consent form and a copy of the participant information sheet will be retained in the investigator’s archive. The participant will receive the information sheet and a copy of the signed consent form.
